# *Caenorhabditis elegans* as a Model System for Studying Drug Induced Mitochondrial Toxicity

**DOI:** 10.1371/journal.pone.0126220

**Published:** 2015-05-13

**Authors:** Richard de Boer, Ruben L. Smith, Winnok H. De Vos, Erik M. M. Manders, Stanley Brul, Hans van der Spek

**Affiliations:** 1 Molecular Biology & Microbial Food Safety, Swammerdam Institute for Life Sciences (SILS), Faculty of Science (FNWI), University of Amsterdam, Science Park 904, 1098 XH, Amsterdam, The Netherlands; 2 Cell Biology and Histology Group, Department of Veterinary Sciences, University of Antwerp, Groenenborgerlaan 171, 2020, Antwerp, Belgium; 3 Cell Systems and Imaging Research Group, Department of Molecular Biotechnology, Ghent University, Coupure Links, 653, 9000, Ghent, Belgium; 4 van Leeuwenhoek Center for Advanced Microscopy, Swammerdam Institute for Life Sciences (SILS), Faculty of Science (FNWI), University of Amsterdam, Science Park 904, 1098 XH Amsterdam, The Netherlands; East Carolina University, UNITED STATES

## Abstract

Today HIV-1 infection is recognized as a chronic disease with obligatory lifelong treatment to keep viral titers below detectable levels. The continuous intake of antiretroviral drugs however, leads to severe and even life-threatening side effects, supposedly by the deleterious impact of nucleoside-analogue type compounds on the functioning of the mitochondrial DNA polymerase. For detailed investigation of the yet partially understood underlying mechanisms, the availability of a versatile model system is crucial. We therefore set out to develop the use of *Caenorhabditis elegans *to study drug induced mitochondrial toxicity. Using a combination of molecular-biological and functional assays, combined with a quantitative analysis of mitochondrial network morphology, we conclude that anti-retroviral drugs with similar working mechanisms can be classified into distinct groups based on their effects on mitochondrial morphology and biochemistry. Additionally we show that mitochondrial toxicity of antiretroviral drugs cannot be exclusively attributed to interference with the mitochondrial DNA polymerase.

## Introduction

Mitochondrial dysfunction is the underlying cause of a wide range of human diseases[1]. In recent years it has become increasingly clear that mitochondrial dysfunction can also be caused by treatment with therapeutic drugs[2]. One important disease in which drug related toxicities have recently been observed is infection with Human Immunodeficiency Virus 1 (HIV-1), the causative agent of Acquired Immunodeficiency Syndrome (AIDS)[3–6].

Highly Active Anti-Retroviral Therapy (HAART) is the latest development in the long quest for a therapy that effectively inhibits viral replication. The introduction of HAART, consisting of a combination of different (classes) of anti-retroviral drugs, has led to a dramatic reduction in HIV-1 related morbidity and mortality[7]. The current therapy guidelines for first-line treatment consist of a combination of two Nucleoside Reverse Transcriptase Inhibitors (NRTIs) combined with a Protease Inhibitor (PI) or a Non-Nucleoside Reverse Transcriptase Inhibitor (NNRTI)[8]. Due to this therapy, at least in developed countries, HIV infection has acquired the characteristics of a chronic disease requiring life-long treatment. However, during the past fifteen years many severe adverse side effects have been reported. These side effects include, among others, fever, rashes (hypersensitivity), respiratory and/or gastrointestinal symptoms, neuropathies, myopathies, nephrotoxicity, lipodystrophy, and hepatotoxicity (for a review see[9]). As an example, body fat distribution is reported to be perturbed in up to 80% of the patients receiving HAART[6]. Additionally, such side effects are the main reason for patients to switch their drug regimen[10]. Hence, it is essential to obtain a better understanding of the molecular mechanisms underlying these adverse effects and to identify novel drugs with fewer side effects.

In humans, the toxic effects of most anti-retroviral drugs strongly resemble those known for hereditary diseases caused by mitochondrial dysfunction[11]. Although it has been shown that PIs also may have side effects on mitochondrial function[9], NRTIs are the prime suspects. The common hypothesis to explain the toxic effect of the NRTIs on mitochondria is frequently referred to as the “DNA polymerase **γ** hypothesis”[3,12]. In essence, NRTIs could interfere with proper mitochondrial (mt) DNA polymerase γ function by causing chain termination, thus resulting in a decline of mtDNA copies. Consequently, the expression of subunits of the Mitochondrial Respiratory Chain (MRC) would also decline, leading to a dysfunctional MRC, illustrated by changes in respiratory rate, a decrease in ATP production, changes in mitochondrial membrane potential, energy depletion and increased ROS production[13,14]. It has indeed been shown that *in vivo* DNA polymerase γ has a high affinity for NRTIs[15]. Nevertheless, it is clear that NRTI induced toxicity not only acts via DNA polymerase γ, but that a more complex relationship between NRTIs and the mitochondria exists[9,16]. To date, most studies have been performed on patients or human cell cultures. Such approaches respectively pose limitations on the experiments that can be performed or limit their physiological relevance for whole organisms. Therefore progress in this field is highly dependent on robust and reliable integrated model systems.

In this study we describe the free-living soil nematode *Caenorhabditis elegans* as versatile model system to investigate the molecular basis of HAART-induced mitochondrial dysfunction. *C*. *elegans* has a proven track record for the elucidation of molecular pathways implicated in human diseases, including diseases with a mitochondrial origin[17–19]. Additionally, it has frequently been used to study mitochondria and drug specific effects[20–25]. Not only is the *C*. *elegans* genome fully sequenced, it is also a very practical system that is easily amenable to genetic modification. It is one of the smallest organisms known to have several types of differentiated tissue such as muscle, dermal, nervous and intestinal tissue and can thus act as a model system for higher eukaryotes. Previous studies clearly underline the usefulness of *C*. *elegans* for assessing mitochondrial function[26,27]. The metabolism and structure of the *C*. *elegans* MRC closely resembles its mammalian counterpart[28]. Moreover, the nematode mtDNA is similar in size and gene content to the human mtDNA. With this in mind we use *C*. *elegans* to evaluate the effects of individual antiretroviral drugs on mitochondrial integrity and function.

In this study, we use a highly sensitive and robust quantitative polymerase-chain-reaction (PCR) assay for mitochondrial DNA in *C*. *elegans*, a functional test (respiration), as well as an automated and quantitative analysis of mitochondrial network morphology. Together they illustrate that *C*. *elegans* is a powerful model system to study the complicated and partially understood side effects that anti-retroviral drugs cause in large numbers of HIV-1 infected patients. The results we present here confirm the notion that the negative effects of NRTIs on mitochondria cannot exclusively be explained by their interference with the functioning of DNA polymerase **γ** by showing that mitochondria can be perturbed by their presence without the concomitant loss of mtDNA.

## Results

### Exposure of *C*. *elegans* to various NRTIs causes severe mtDNA depletion

**Fig 1:**
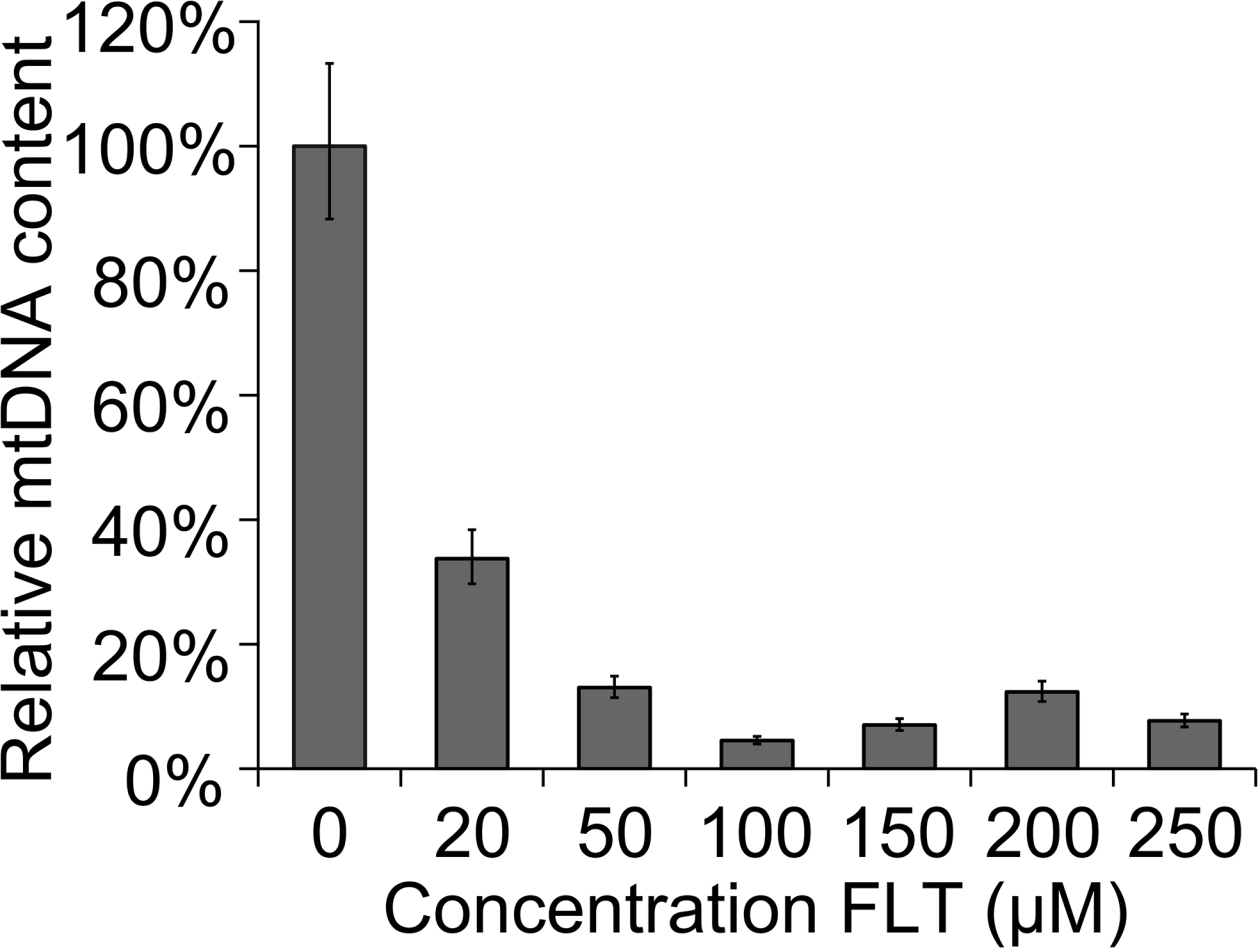
Concentration dependent decrease of mtDNA. Synchronised L1 worms were put on a plate with FLT and experiments were performed after 72hrs of continuous exposure. In FLT exposed animals, the reduction of mtDNA is concentration dependent. Error bars represent 95% CI (df = 16). Significance was determined using a two-tailed student’s T test assuming unequal variances. P-value was <0.001 for all reported concentrations.

In order to validate the use of *C*. *elegans* as a model for drug induced mitochondrial dysfunction we first needed to establish whether the side effects that occur in humans can also be seen when exposing *C*. *elegans* to NRTIs, the backbone of HAART. The most obvious effect of NRTIs in patients is the reduction of mtDNA copy numbers[29]. To assess this in *C*. *elegans* we set up a quantitative PCR assay for mtDNA using the conserved mitochondrial DNA encoded COX1 gene as a target (see Materials and Methods). In a typical experiment, the nematodes were synchronized and consequently exposed to fixed concentrations of NRTIs for 72 hrs. Drugs were either mixed in with the bacterial lawn or added directly to the agar plates. Both methods were effective and are reported previously[30,31]. Since no data was available for uptake and sensitivity of *C*. *elegans* for this class of drugs we started with the NRTI 3’-deoxy-3’-Fluorothymidine (alovudine or FLT) as a benchmark. FLT is no longer used to treat HIV-1 infected individuals because of its well-known severe mitochondria related toxicity in patients[32]. Exposing the nematodes to FLT resulted in a significant decrease in mtDNA copy number (Fig 1). This decline is concentration dependent, starting from an absolute control value of 3,8x10^6^ copies per worm. Maximal reduction to 10% of the control was reached at a concentration of 100 μM FLT. Relative quantification of mtDNA copies per nDNA (qPCR of the nuclear actin gene as described previously[33] gave identical results (S1 Table).

**Fig 2:**
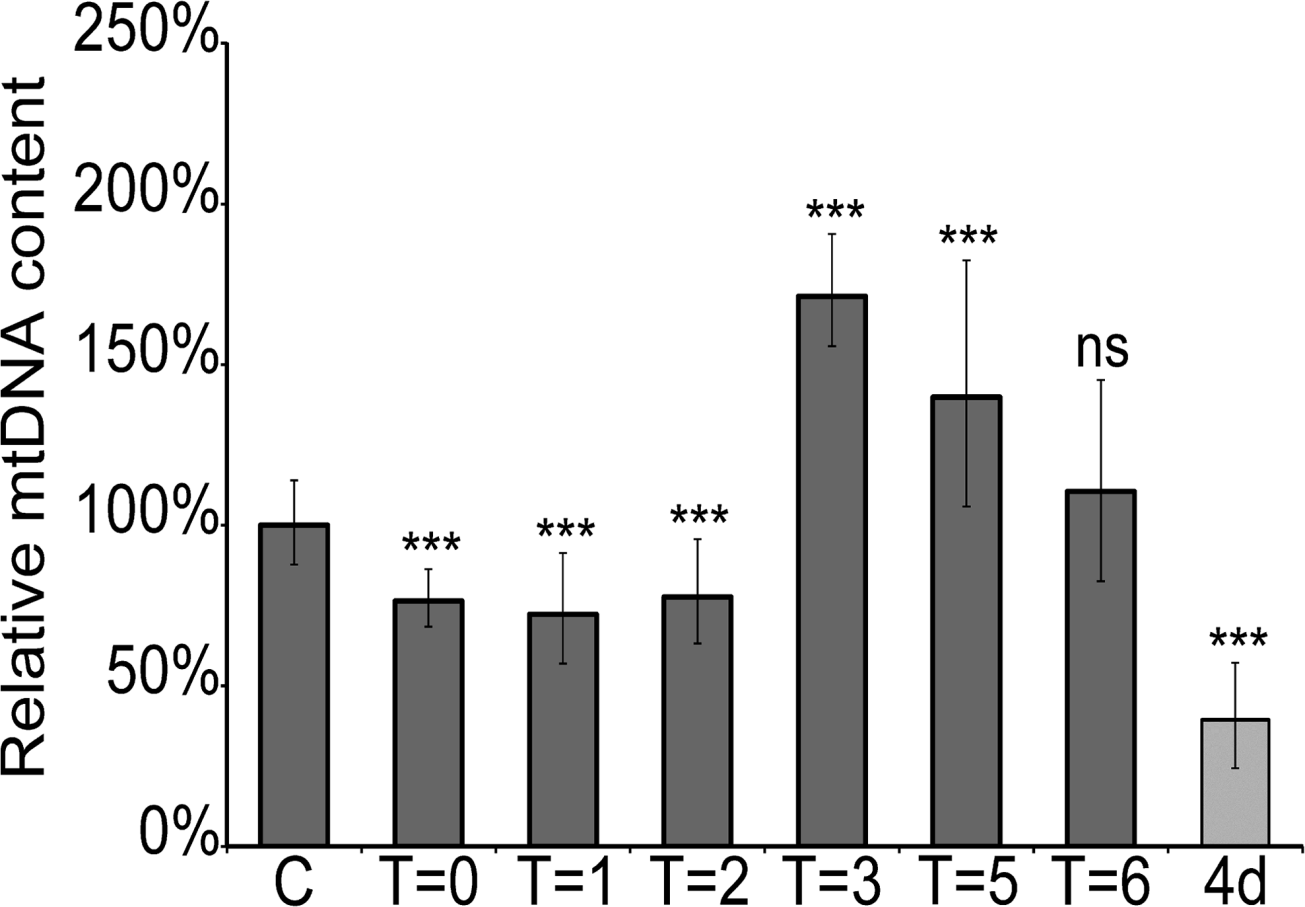
Recovery of mtDNA after cessation of exposure. Worms are exposed to FLT (200 μM) from D1 of adulthood onwards for 72hrs after which they are transferred to plates without FLT. This results in a recovery of mtDNA. Error bars show the 95% C.I. (df = 16). C = untreated worms, T = #hr after transfer, 4d = 4days of continuous exposure. Significance was determined using a two-tailed student’s T test assuming unequal variances. ***P-value <0.001 compared to unexposed animals. ns: not significant compared to unexposed animals.

In clinical practice the mitotoxic effects of NRTIs on patients can mostly be reversed if the treatment is interrupted [34]. To examine whether the mtDNA lowering effect of FLT is also reversible in *C*. *elegans* we performed an experiment in which adult nematodes were first exposed to 200μM FLT for 72 hrs to be certain that the maximum effect was obtained, after which the worms were transferred to a fresh plate without FLT. Adult worms were exposed to avoid any effects on the development of the worms that might influence the results. Samples were taken after 1, 2, 3, 5 and 6 hours after transfer and analyzed for mtDNA content. The results depicted in Fig 2 clearly demonstrate that FLT induced mtDNA depletion is reversible. The first two hours after transfer there is no significant recovery of mtDNA levels. Surprisingly, after three hours the mtDNA level is significantly elevated compared to pre-exposure levels. After 5 and 6 hours the amount of mtDNA gradually declines to normal levels. This effect suggests that during exposure to NRTIs, the mitochondria try to compensate for loss of mtDNA.

In patients not all NRTI drugs cause the same level of mtDNA decrease[29]. In order to see whether this is also observed in *C*. *elegans*, worms were exposed to a selection of frequently used NRTIs. It has been shown that DNA polymerase **γ** has an affinity for all NRTIs[9,35]. However, unlike FLT, none of the other NRTI’s induced mtDNA reduction at 100μM concentration after 72 hrs (data not shown). We therefore repeated the experiment using higher concentrations at prolonged incubation times (up to 192 hrs, Table 1). The results show that with these concentrations, mtDNA reduction is observed after 96 hrs incubation with 3’-azido-3’-deoxythymidine (Zidovudine or AZT), 2'-3'-didehydro-2'-3'-dideoxythymidine (Stavudine or d4T), 2',3'-dideoxyinosine (Didanosine or ddI) and 2'-3'-dideoxycytidine (Zalcitabine or ddC). The reduction of mtDNA with these NRTIs is clearly modest compared to exposure to FLT. NRTI MIC values on *E*. *coli* OP50 were determined and with the exception of AZT, no growth perturbation was found at relevant concentrations. (S2 Table) The growth inhibitory effect of AZT[36] has no visible influence on nematode physiology and drug properties remain consistent when administered with UV deactivated *E*. *coli*, as measured by mtDNA copy number (S3 Table).

**Table 1:** Relative quantities of mtDNA compared to unexposed animals.

	72h (+/-)	96h (+/-)	120h (+/-)	144h (+/-)	168h (+/-)	192h (+/-)
**Control**	100% (9/10)	100% (12/11)	100% (12/11)	100% (24/21)	100% (13/12)	100% (10/9)
**AZT 400 μM**	60%^*^ (6/7)	40%^***^ (5/4)	114%^ns^ (10/6)	53%^***^ (6/6)	63%^***^ (8/7)	40%^***^ (5/4)
**AZT 800 μM**	105%^ns^ (9/10)	48%^***^ (6/5)	45%^***^ (8/7)	121%^*^ (10/9)	61%^***^ (5/5)	75%^**^ (6/6)
**D4T 400 μM**	28%^***^ (3/3)	25%^***^ (3/3)	134%^**^ (9/8)	52%^***^ (6/6)	74%^**^ (9/8)	44%^***^ (5/5)
**D4T 800 μM**	43%^***^ (4/5)	44%^***^ (5/5)	55%^***^ (7/6)	68%^***^ (10/9)	30%^***^ (6/6)	62%^***^ (7/6)
**FLT 400 μM**	4%^***^ (0/1)	3%^***^ (0/0)	8%^***^ (1/1)	5%^***^ (1/1)	9%^***^ (1/1)	4%^***^ (1/0)
**FLT 800 μM**	6%^***^ (1/1)	5%^***^ (1/0)	4%^***^ (1/1)	4%^***^ (1/1)	2%^***^ (1/1)	6%^***^ (1/1)
**ddI 400 μM**	81%^ns^ (9/10)	42%^****^ (5/4)	97%^ns^ (11/10)	74%^**^ (9/8)	75%^**^ (9/8)	79%^*^ (9/8)
**ddI 800 μM**	130%^**^ (11/13)	70%^***^ (8/7)	99%^ns^ (9/7)	105%^ns^ (14/12)	63%^***^ (6/6)	131%^**^ (13/11)
**ddC 400 μM**	84%^ns^ (9/10)	50%^***^ (6/5)	121%^*^ (14/13)	78%^*^ (9/8)	96%^ns^ (11/10)	65%^***^ (8/7)
**ddC 800 μM**	102%^ns^ (12/13)	55%^***^ (6/6)	97%^ns^ (11/10)	100%^ns^ (15/13)	74%^**^ (8/7)	80%^*^ (10/9)

P-value was determined compared to unexposed animals. Significance was determined using a two-tailed student’s T-test assuming unequal variance. Numbers between parentheses indicate 95% confidence intervals (16df)

* p-value <0.05

** p-value <0.01

*** p-value <0.001

ns: not significant.

### O_2_ consumption rate is affected by exposure to some NRTIs

To investigate whether mtDNA depletion also affects mitochondrial respiratory chain functioning, oxygen consumption was measured after 72hr of exposure to NRTIs (100μM).

**Fig 3:**
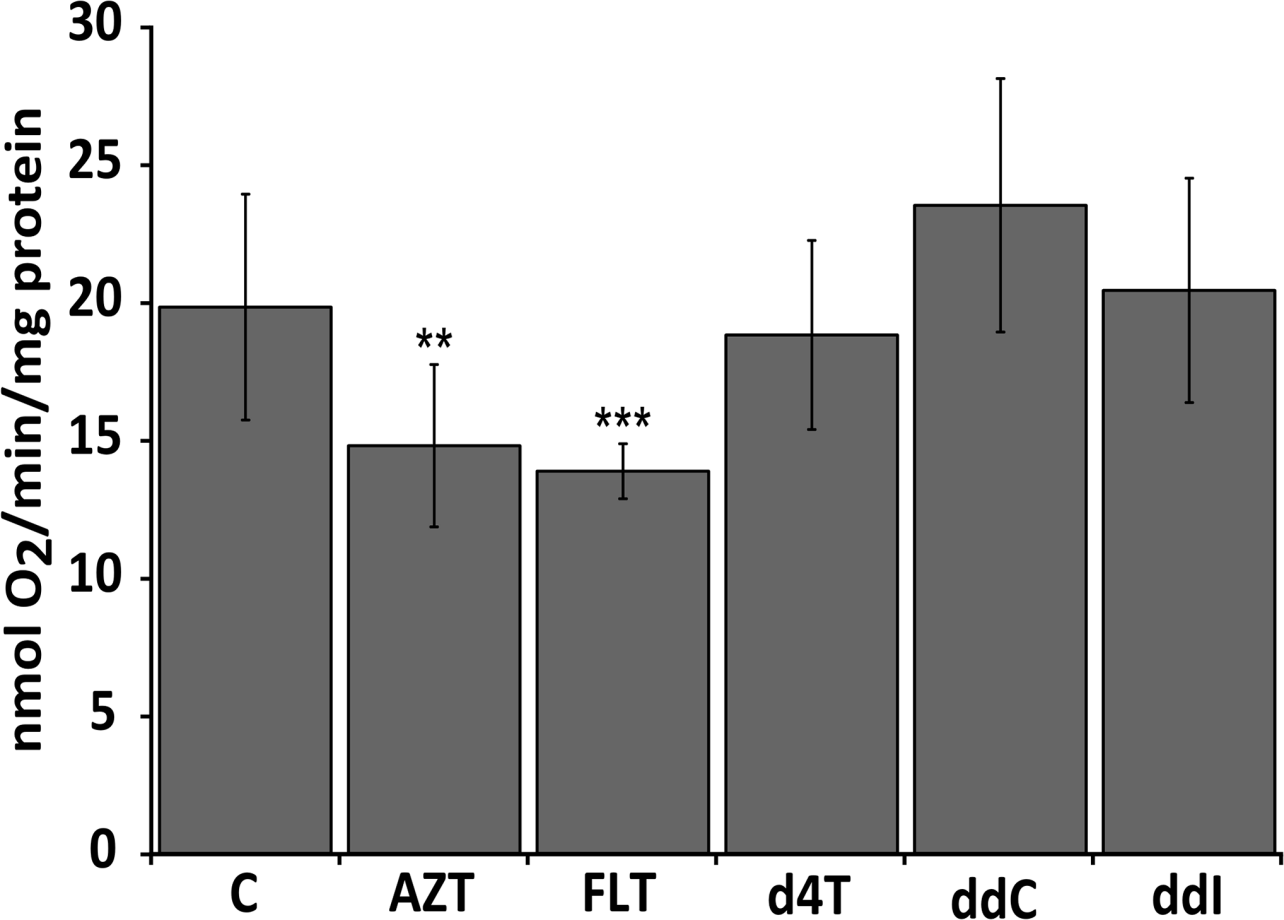
Oxygen consumption rates in NRTI exposed worms. Drug concentration was 100μM in all experiments. FLT and AZT exposed worms show a significantly reduced oxygen consumption rate compared to unexposed animals. C = Control, ** p-value <0.01, *** p-value <0.001. Significance was determined using a two tailed student’s t-test assuming unequal variance.

Whereas FLT and AZT induce a significant reduction of oxygen consumption, no significant effects were observed after treatment with d4T, ddC or ddI (Fig 3). For d4T, ddC and ddI these results correlate well with the lack of mtDNA depletion seen with these NRTIs at this concentration and incubation time (Table 1). In the case of AZT treated worms however, a significant decrease in oxygen consumption can be observed despite the absence of mtDNA depletion with this NRTI (Table 1).

### Quinone redox state is altered by some NRTIs

*C*. *elegans* mitochondrial coenzyme Q (CoQ9) serves as a mobile electron carrier in the mitochondrial respiratory chain where it shuttles electrons from complex I and II to complex III. The ratio between reduced (ubiquinol) and oxidized (ubiquinone) forms of CoQ9 indicate that mitochondrial respiratory chain enzyme complexes may have reduced activity, and has been proposed as a useful biomarker for diagnosing and managing patients with mitochondrial respiratory chain defects[37]. To assess the effects NRTIs have on mitochondrial respiratory enzyme complex activity, CoQ9 redox ratio was measured after 72hr of exposure (100μM).

**Fig 4:**
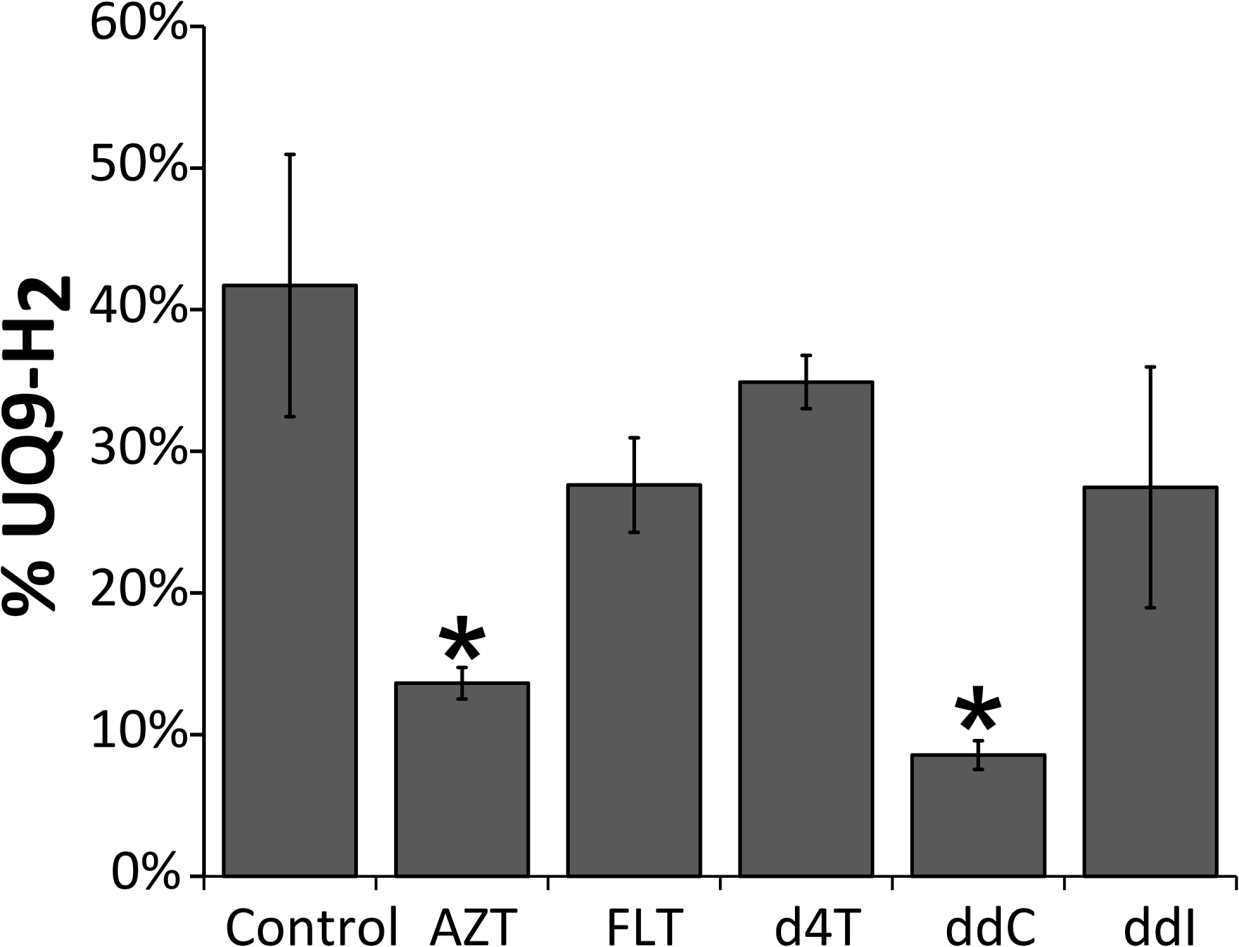
Quinone redox status is altered by some NRTIs. The redox state of the UQ_9_ pool is presented as percentage reduced (i.e. UQ_9_-H_2_) of the total UQ_9_ pool. Error bars indicate standard error. * = P ≤ 0,05.

AZT and ddC treated worms show significantly lower levels of reduced CoQ9, whereas no significant effects were seen for FLT, d4T and ddI (Fig 4). For AZT these results are in line with diminished oxygen consumption (Fig 3) as animals with inhibited electron shuttling are likely to consume less oxygen.

### Exposure to NRTIs causes differential alteration of mitochondrial ultrastructure

**Fig 5:**
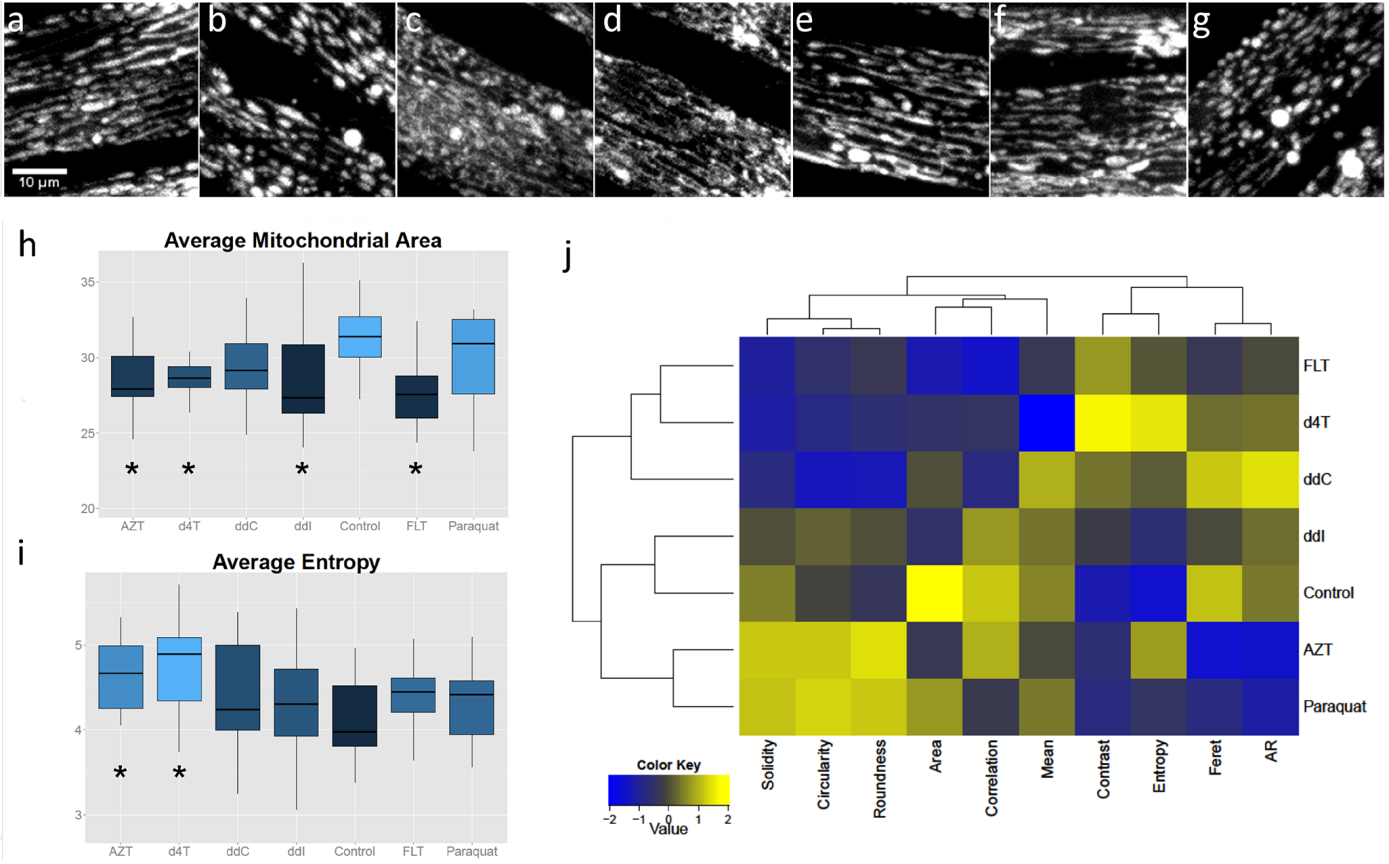
Exposure to NRTIs causes changes in mitochondrial morphology. a-g: Mitochondrial morphology in the body wall muscle of worms exposed to NRTIs (200 μM) (a: control, b: AZT, c: FLT, d: d4T, e: ddI, f: ddC) and g: Paraquat.) (h,i): Boxplots comparing selected metrics per condition. Asterisks indicate statistically different from control (per metric). (j): Two-dimensional clustergram performed on the standardized dataset (z-scores) of features extracted after image analysis, using Euclidean distance as distance metric and the average value as linkage value for the dendrograms

It has been shown that mitochondrial fusion is required for mtDNA stability[38] and mitochondrial morphology can be influenced by exposure to antiretroviral drugs[39]. To visualize these effects in *C*. *elegans* we used a transgenic *glo-1(zu391)* strain with a mitochondrially localized GFP marker which is regulated by the muscle-specific *myo-3* promoter[40]. A quantitative morphological comparison of the mitochondrial network in anterior muscle cells of *C*. *elegans* revealed that some NRTIs cause a significant disruption of the mitochondrial network (Fig 5A–5G) in comparison with DMSO as control. Some drugs induce more complex, tubular mitochondrial networks (FLT, d4T and ddC; Fig 5C, 5D and 5F). Other drugs induce fragmented, blob-like structures (AZT, Paraquat; Fig 5B and 5G). The results for ddI (Fig 5E) are most comparable-but not identical- to the control phenotype. Interestingly, FLT, the drug with the most significant effect on mtDNA copy number, shows a decrease in mitochondrial area, similar to worms deficient of DNA polymerase γ[33]. For several single parameters drug-induced effects are clearly different from the control, like the decreased area induced by FLT (Fig 5H) and the increase in average entropy induced by AZT and d4T (Fig 5I). In an integrative approach, exhaustive data extraction of all parameters and subsequent hierarchical clustering of all data (2-dimensional clustergram Fig 5J) showed a clear segregation of drug treatments into two distinct classes that differ from the control phenotype which shows a semi-aligned network of mitochondria of intermediate length (DMSO treated; Fig 5A). Remarkably, despite the fact that ddC and d4T do not result in reduced mtDNA levels they do cluster together with FLT. In contrast, AZT which has no effect on mtDNA content does show a clear altered morphological effect with a fragmented, blob-like mitochondrial phenotype.

## Discussion

Since the introduction of a successful therapy in 1996, HIV-1 infection has turned into a chronic disease. However, it has become painfully clear that the obligatory lifelong intake of antiretroviral drugs comes with a heavy burden: severe and even life-threatening side effects. For detailed investigation of the yet poorly understood underlying mechanisms the availability of a versatile model system is crucial. Here we describe the deployment of *C*. *elegans* as such a model. Our results show that *C*. *elegans* is an excellent and accessible model-system to study drug induced mitochondrial toxicity.

First, a quantitative PCR assay for *C*. *elegans* mtDNA was developed since mtDNA depletion has been observed frequently in patients treated with antiretroviral drugs. Similar effects in *C*. *elegans* would be a strong indication that the drugs enter the worm cells and act in a similar way as in human cells. Using the COX1 gene as a target we were able to set up a very robust and reproducible RT-PCR. Using this assay we showed that different NRTIs result in a reduction of mtDNA copy number, albeit to a variable degree (Table 1). We also demonstrated that depletion of mtDNA is NRTI concentration dependent and reversible (Figs 1 and 2) (Note: the difference in FLT induced mtDNA depletion between the two figures is due to the fact that in Fig 1 the worms were exposed from L1, and in Fig 2 72 hr after adulthood). An interesting observation is the overshoot of mtDNA after transfer of the worms to drug-free plates (Fig 2). This suggests that compensatory mechanisms may operate during exposure to NRTIs in an attempt to keep mtDNA content at a sufficient level that is compatible with life e.g. by overproduction of the replication machinery resulting in a transient overshoot/overproduction of mtDNA upon removal of the inhibitor. Interestingly, this result closely matches a report of a patient receiving HAART[34], fortifying the usefulness of *C*. *elegans* as a model system for drug induced mitochondrial toxicity.

Second, to investigate whether a decline in mtDNA correlates with a reduction of mitochondrial function we tested O_2_ consumption rates. O_2_ consumption rate was significantly reduced in *C*. *elegans* treated with the drug that resulted in the strongest mtDNA reduction (FLT; Fig 3). In addition, also worms exposed to AZT showed a strongly reduced O_2_ consumption (Fig 3). This result is unexpected since mtDNA is not reduced at the concentration and incubation period used. No reduced O_2_ consumption is observed with the other drugs that do not reduce mtDNA levels (d4T, ddI, ddC).

Third, the results obtained with AZT underline that mtDNA depletion per se is not a prerequisite for mitochondrial dysfunction, another explanation could be interference with the electron transport chain. Indeed, CoQ9 redox state was significantly altered by AZT and ddC (Fig 4). More reduced CoQ9 pools for AZT correlates with reduced oxygen consumption. ddC interestingly shows neither lower mtDNA copy numbers or oxygen consumption. A reduction of mitochondrial respiratory chain complex I activity for AZT and an inhibited phosphorylation of complex I by ddC, however, has previously been described. Altered phosphorylation of complex I Q-site subunits may affect NADH:CoQ oxidoreductase activity and lead to a more reduced quinone pool[41].

Finally, keeping in mind the intricate relationship between mitochondrial function and morphology, we analyzed the morphology of the mitochondrial network in muscle cells of *C*. *elegans* by confocal fluorescence microscopy. Mitochondrial fusion, fission and autophagy are important for protection against persistent mtDNA damage[38,42]. Therefore, altered mitochondrial morphology might be considered as a marker of mitochondrial health as well as a compensatory mechanism in helping to preserve mitochondrial functions. Conversely, mitochondrial dysfunction may be reflected in altered morphology. We found significant differences between the tested drugs, with two major, opposing, phenotypical effects on the mitochondrial network structure; either yielding more tubulated (FLT, d4T and ddC) or fragmented (AZT) networks. This is a highly interesting result, since the effect of AZT on morphology mimics the effect of Paraquat, a compound known to disrupt mitochondrial morphology. Paraquat is also well known for its strong induction of reactive oxygen species[43]. ddI exposure results in a near-normal phenotype, comparable to the control incubation (worms exposed to DMSO, the solvent used for NRTIs).

Taken together, these data suggest that mitochondrial function can be affected by NRTIs in a mtDNA polymerase γ–independent way. In humans, ddC is regarded as one of the most toxic NRTIs, resulting in limited prescription in recent years[44]. In post-mitotic cells, mitochondria rely partly on salvage pathways by deoxynucleotide kinases. *C*. *elegans* appears to have only one cytoplasmic salvage kinase (THK-1) displaying high activity with thymidine, but low or no activity with deoxyguanosine, deoxycytidine and deoxyadenosine [45]. It is therefore expected that ddC and ddI will not be phosphorylated, suggesting that mtDNA depletion observed with these drugs is either non-specific or due to interference with other steps in nucleotide metabolism. Preliminary RNAi experiments have shown that when THK-1 is knocked down, exposure to FLT (200uM) but not AZT (200uM) leads to loss of reproduction, indicating an excerbation of the toxic effects with the unphosphorylated form of the thymidine analogue FLT (results not shown). This underlines our hypothesis that chain termination in mtDNA replication by the triphosphorylated form is not the only mechanism involved in NRTI toxicity. In light of this, our observations stress the usefulness of independent and separate parameters as indicators of unwanted effects on mitochondria by anti-HIV-1 medication and give indications for different mechanisms behind the toxicity of these different compounds. Moreover, these findings underscore the fact that there is more to NRTI toxicity than the interaction with DNA polymerase γ alone[9]. One possible interpretation of our results could be that without detectable mtDNA depletion (AZT), inhibition of the mitochondrial respiratory chain complexes as indicated by more reduced CoQ9 pools causes increased radical formation and fragmented mitochondrial networks. Also, with normal oxygen consumption levels (d4T, ddC) morphological changes in the mitochondria (as reflected by the tubular structures) are observed, comparable to FLT exposure. With strong mtDNA depletion (e.g. using FLT), oxygen consumption (and likely ATP generation) undergo a sharp drop on top of the earlier problems.

In conclusion, we show here that *C*. *elegans* can be used as an excellent model system to further unravel the mechanistic basis of observed adverse effects of both individually and collectively administered antiretroviral drugs. Besides the deleterious effects of individual drugs, a highly relevant issue is the effect of drug combinations. This model system will allow systematic screening of different combinations of drugs resulting in a better prediction of preferred or less-preferred combinations. As an example, it has been shown already that the protease inhibitor Ritonavir interferes with the efflux pump cytochrome P-450, thereby increasing the intracellular concentrations of other drugs[46]. Of crucial importance, this system can also be used to screen novel therapeutic compounds with less unwanted side effects as well as to probe for molecules that are able to alleviate detrimental effects. Thus the *C*. *elegans* model that we describe allows for a more comprehensive screen for mitochondrial toxicity of NRTIs and will thus provide unique opportunities to improve drug-treatment regimens and thereby help to improve quality of life for many patients.

## Materials and Methods

### Strains and conditions

Worms were cultured at 20°C and fed with *E*. *coli* OP50 as a food source, unless otherwise noted. *C*. *elegans* strains used were N2 Bristol wild-type strain and *glo-1(zu391)*. Exposure to drugs was performed by adding the drugs to the bacterial lawn or agar plate to a final concentration as mentioned.

### Drugs

Dimethylsulfoxide (D4540 Sigma-aldrich), 2′,3′-Didehydro-3′-deoxythymidine (D1413 Sigma-aldrich), 3′-Azido-3′-deoxythymidine (A2169, Sigma-aldrich), 2'-3'-dideoxycytidine (D5782, Sigma-Aldrich), 2',3'-dideoxyinosine (D0162, Sigma-Aldrich), 3’-deoxy-3’-Fluorothymidine (361275, Sigma-Aldrich), Methyl viologen dichloride hydrate (856177 Sigma-Aldrich).

### Real time PCR

The mtDNA copy number was measured using quantitative Real Time PCR. Exposure was performed by adding the drugs to the OP50 before spreading it on the plates. Synchronized worms were placed on the plates and allowed to develop for 72 hours after which five adult worms were collected and lysed (Lysis buffer: 50mM KCl, 10mM Tris (pH 8.3), 2.5mM MgCl2, 0.45% NP-40 (IGEPAL),0.45% Tween-20, 0.01% Gelatin, 0.1mg/mL proteinase K). Before detection in the PCR, the solution was diluted 40 times and 2 μl was used as input in the PCR reaction. Primers specific for cytochrome c oxidase subunit I (COX1) were used for the determination of mtDNA copy number. Ce*COX1* Forward primer: 5’-GGGCTATTACTATGTTGTTAACTGATCGT-3’. CeCOX1 reverse primer: 5’-AAATCAAAATATATACTTCAGGATGACCA-3’. PCRs were performed using the Taqman universal cycling conditions. Amplified products were detected using a Taqman probe: CeCOX1: 6-FAM-ACTTCATTTTTTGATCCAAGAACTGGAGGTAATCCT-TAMRA and the TaqMan Universal PCR Master Mix with ROX, (Applied Biosystems). Primer concentration in each reaction was 300nM and probe concentration was 250nM. Fluorescent signal intensities were determined using the 7300 Real-Time PCR System (Applied Biosystems) with software SDS (version 1.9.1). To measure the mtDNA copy numbers, Ct values were determined using the linear exponential phase from a standard curve generated by using plasmid containing cloned target sequence (COX1) into the pUC19 plasmid. Absolute values were determined by 7 tenfold dilutions of plasmid DNA with known concentrations. Quantitative PCR was performed at least four times and the results were reproducible. Primers were tested for specificity using a SYBR green assay and melting curve analysis. Five worms were picked and analyzed for mtDNA content by means of a quantitative PCR. After PCR the total mtDNA copies per worm were calculated. Quantification of nDNA has been performed and showed no difference between conditions (S1 Table).

### Relative quantification qPCR

The mtDNA was quantified using the following method. First a dilution series was prepared and quantified in triplicate. Using the determined values a regression line was plotted using Microsoft Excel’s Linreg function. Next, ct values of the unknown samples were averaged and linear regression was performed to determine the 95% confidence interval. Finally the absolute values were normalized to the unexposed reference samples.

Significance was determined using a t-test with $H_{0}=\bar{x_{1}}=\bar{x_{2}}$ and the t-value was calculated using $t=\frac{\left(\bar{x_{1}}=\bar{x_{2}}\right)}{s_{p}\sqrt{\frac{1}{n_{1}}+\frac{1}{n_{2}}}}$: and $s_{p}=\sqrt{\frac{\left(n_{1}-1\right)s_{1}^{2}+\left(n_{2}-1\right)s_{2}^{2}}{n_{1}+n_{2}-2}}$. n_1_ and n_2_ are the number of observations in the two groups. S_p_ is the pooled standard deviation. A F-test was performed to check whether the standard deviations were statistically different.

### Oxygen consumption measurements

Oxygen consumption rates were measured using a Neofox fiber optic oxygen sensor (Ocean optics). Synchronized D1 adult worms were kept on 50μM of 5’-Fluoro-2’-deoxyuridine (FUdR, 46875 Fluka) and exposed to 100μM of drugs mixed in the NGM medium for 72h, washed three times with S-basal to remove any bacteria and pelleted. 100μL of worm pellet (~ 1mg protein) was suspended in 1mL O_2_ saturated S-basal in an oxygraph chamber and oxygen concentration was measured for a minimum of 10 minutes. The slope of the straight portion of the plot was used do derive the oxygen consumption rate. Worms were recovered after respiration measurements and collected for protein quantification using the Lowry method. Rates were normalized to protein content as previously described[47].

### Quinone extraction & redox state measurements

Approximately 4000 wild type worms were cultured on NGM plates seeded with *menA* ^*-*^ *E*. *coli* until young adult stage. MenA is an essential protein in the menaquinone biosynthesis of *E*. *coli*. *menA* ^*-*^ was used because menaquinone-8 interferes with the UQ_9_ HPLC signal[48]. Young adult worms were transferred to NGM control plates or plates containing 100μM drug and cultured for 72 hours. Worms were transferred daily to fresh plates after washing with M9 buffer and filtered through a Nitex μM filter (03-31/24, Sefar AG Filtration Solutions, Heiden, Switzerland) to rid culture of larvae and eggs. After 72h of culture worms were washed from plates with ice-cold M9 buffer, filtered and collected in 8mL ice-cold MeOH/PCa (60:1). 1mL of worm suspension was separated and used for Lowry protein measurements (1mg protein). Quinones were extracted from the remaining worm suspension by pottering on ice with the Potter S (Satorius, type: 8533024) (20x @ 1000revs/min) to ensure complete lysis of cells whilst flushing the potter tube with N_2_. Next 6mL of analystical grade petroleum ether (40–60^°^C) was rapidly added to the suspension and vortexed for 1 minute. After the mixture was centrifuged (1 minute, 3000rpm) the upper petroleum ether phase was removed, transferred to a test tube and evaporated to dryness under a flow of nitrogen. Another 6mL of petroleum ether was added to the remaining suspension, whereafter extraction was repeated. After evaporation to dryness extracts were stored for maximally 9 days under nitrogen at -20^°^C before being analyzed.

Immediately before use, the extracts of UQ/ubiquinol were resuspended with a glass rod in 60 μl ethanol and analysed by HPLC, using a Pharmacia LKB gradient pump 2249 system. The instrument was equipped with a fluorescence detector (Agilent 1260 infinity FLD) and a reverse-phase Lichrosorb (Chrompack) 10 RP 18 column (4.6-mm i.d., 250-mm length) at 50^°^C. The column was equilibrated with pure methanol which was also used as the mobile phase. The flow rate was set at 2 ml min^−1^. Detection of the quinones was performed at 290/248 nm and peaks were identified by UV/Vis spectral analysis at 12 and 20 minutes for UQ_9_-H_2_ and UQ_9_ respectively. Methanol, ethanol and petroleum ether were of analytical grade (Sigma). The redox state of the UQ_9_ pool is presented as percentage reduced (i.e. UQ_9_-H_2_) of the total UQ_9_ pool. A minimum of three biological replicates were analysed. A one-sided students t-test assuming unequal variance was used to asses significance.

#### Image acquisition and analysis of mitochondrial morphology

Mitochondrial morphology in body wall muscle cells was visualized in transgenic *glo-1(zu391)* animals using mito::GFP expressed from the *myo-3* promoter making it ideal for the analysis of mitochondrial morphology. Synchronized worms were allowed to develop until L4, after which they were transferred to plates with drugs added to the NGM. 24h later at least 10 individual worms were imaged using the Nikon A1 confocal microscope, with a Plan Apo 60x WI objective with a numerical aperture of 1.27, a 488nm argon laser, a pixel size of 0,2μM and NIS-Elements AR v4.1004 (Build 854) software. Image processing was performed in ImageJ freeware (W.S. Rasband, U.S.A. National Institutes of Health, Bethesda, Maryland, USA, http://rsb.info.nih.gov/ij/, 1997–2012). Mitochondria were segmented by means of a custom-designed image-processing pipeline (available at www.limid.ugent.be/downloads). First, uninformative slices or slices with reflections were removed from the image stacks, using an image quality criterion that only retains slices with a covariance >1. The triaged stacks were then projected according to the maximum pixel intensity and preprocessed by background subtraction (rolling ball radius 15) and local contrast enhancement (block size 15) after which objects were enhanced by means of a multi-scale Laplacian operator[49]. Subsequently, the image was binarized according to a Yen autothresholding procedure and the resulting mask was used for analyzing shape and intensity metrics of objects larger than a predefined size (>12 pixels) on the original image. In addition, global texture metrics were measured on the original image using the GLCM texture plugin (by Julio Cabrera).

For unsupervised clustering and heatmap generation, a subset of mitochondria- and image-specific descriptors was retained. Of the segmented mitochondria, average values were calculated for Mean (average grey value), Area (average projected area), AR (aspect ratio of the fitted ellipse, i.e. major axis/minor axis), Feret (longest internal distance), Solidity (area/convex area), Circularity (4*pi*area/perimeter^2^) and Roundness ((4*area)/(pi*major axis^2^)). Of the texture parameters, the following metrics were calculated as averages from a horizontal and a vertical gray-level co-occurrence matrix (GLCM), both with one pixel offset.: Entropy($\sum_{i,j=0}^{N-1}P_{ij}\left(-\ln P_{ij}\right)$, Contrast ($\sum_{i,j=0}^{N-1}P_{ij}{\left(i-j\right)}^{2}$) and Correlation ($\sum_{i,j=0}^{N-1}P_{ij}\left\lfloor \frac{\left(i-\mu_{i}\right)\left(i-\mu_{j}\right)}{\sqrt{\sigma_{i}^{2}\sigma_{j}^{2}}}\right\rfloor$),with P_i,j_ reflecting values of row i and column j in the GLCM.

### Statistical analysis

Statistical analyses were performed in Microsoft excel 2010, Matlab 2010a (Mathworks, Eindhoven, The Netherlands) or R freeware. Individual parameters (shape and texture descriptors) were statistically compared between conditions, by means of pairwise student’s T-tests or, in case of non-normal distributions, Wilcoxon rank sum tests. Results were summarized in boxplots. K-means cluster analysis was performed on the standardized dataset (z-scores), using Euclidean distance as distance metric and the average value as linkage value for the dendrograms. The results were represented in a two-dimensional clustergram.

## Supporting Information

S1 Table Ct values of QPCR used to quantify the nDNA in NRTI exposed worms. Results show no significant differences between ct values. When absolute values are calculated using the determined regression line formula y = −3.316x + 38.19, results are comparable to previously described results[50]. Significance was determined using a two-sided students t-test assuming equal variance on the obtained ct values of at least five independent replicates.
(DOCX)

S2 Table NRTI MIC values in μM for *B*. *subtilis* 168, *E*. *coli* LMC500 and *E*. *coli* OP50. Only AZT inhibits *E*. *coli* growth at concentrations used in this study. The growth inhibitory effect of AZT has no visible influence on nematode physiology and drug properties remain consistent when administered with UV deactivated *E*. *coli*, as measured by mtDNA copy number (S3 Table).
(DOCX)

S3 Table UV inactivated *E*. *coli* does not alter the effect of AZT on mtDNA copy number. Ct values obtained for mtDNA quantification in *C*. *elegans* exposed to AZT and fed on UV-killed *E*. *coli*. Results show no significant differences between ct values. Significance was determined using a two-sided students t-test assuming equal variance on the obtained ct values.
(DOCX)
