# Supplementary material for: Caenorhabditis elegans as a Model System for Studying Drug Induced Mitochondrial Toxicity
Source: PLoS One. 2015 May 13;10(5):e0126220. doi: 10.1371/journal.pone.0126220 (PMC4430419; doi:10.1371/journal.pone.0126220)
Supplement: S1 Table — Results show no significant differences between ct values. When absolute values are calculated using the determined regression line formula y = −3.316x + 38.19, results are comparable to previously described results[50]. Significance was determined using a two-sided students t-test assuming equal variance on the obtained ct values of at least five independent replicates. (DOCX) [file pone.0126220.s001.docx]

**Table S1. Ct values of QPCR used to quantify the nDNA in NRTI exposed worms.**

|  | Ct value | Stdev | P-value |
| --- | --- | --- | --- |
| Control | 30.36 | 1.42 | - |
| 100µM AZT | 30.91 | 2.14 | ns |
| 100µM FLT | 29.81 | 1.14 | ns |
| 100µM d4T | 30.62 | 1.22 | ns |
| 100µM ddI | 29.66 | 1.20 | ns |

Methods: Nuclear DNA (nDNA) copy numbers were quantified using real time PCR. Primers specific for the actin gene are previously described and were used for the determination of nDNA copy number. CeAct-1 Forward primer: 5’-TGCGACATTGATATCCGTAAGG-3’. CeAct1 reverse primer: 5’- GGTGGTTCCTCCGGAAAGAA -3’. PCRs were performed using the Taqman® universal cycling conditions. Amplified products were detected using the SYBR® Green PCR Master Mix (Applied Biosystems). Primer concentration in each reaction was 300nM. Fluorescent signal intensities were determined using the 7300 Real-Time PCR System (Applied Biosystems) with software SDS (version 1.9.1) To measure the mtDNA copy numbers, Ct values were determined using the linear exponential phase from a standard curve generated by using plasmid containing cloned target sequence (*Act-1*) into the pUC19 plasmid. Absolute values were determined by 7 tenfold dilutions of plasmid DNA with known concentrations. Quantitative PCR was performed at least four times and the results were reproducible. Primers were tested for specificity using a SYBR® green assay and melting curve analysis.
