# Supplementary material for: Caenorhabditis elegans as a Model System for Studying Drug Induced Mitochondrial Toxicity
Source: PLoS One. 2015 May 13;10(5):e0126220. doi: 10.1371/journal.pone.0126220 (PMC4430419; doi:10.1371/journal.pone.0126220)
Supplement: S2 Table — The growth inhibitory effect of AZT has no visible influence on nematode physiology and drug properties remain consistent when administered with UV deactivated E. coli, as measured by mtDNA copy number (S3 Table). (DOCX) [file pone.0126220.s002.docx]

**Table S2.** NRTI MIC values in µM for *B. subtilis* 168, *E. coli* LMC500 and *E. coli* OP50.

|  | B.subt. 168 | E.coli LMC500 | E.coli OP50 |
| --- | --- | --- | --- |
| FLT | ≥ 600 | 67 | 600 |
| AZT | ≥ 600 | ≤ 2,5 | ≤ 2,5 |
| d4T | ≥ 600 | 200 | ≥ 600 |
| ddI | ≥ 600 | 67 | 200 |
| ddC | nd | nd | ≥ 600 |

Methods: NRTI susceptibility testing was performed using *Bacillus subtilis* 168, *Escherichia coli* LMC500 and *Escherichia coli* OP50 strain cultures. Cultures in exponential phase were diluted to an OD of 0.0001 in LB medium and cultured overnight (~14h) at 37ᵒC whilst exposed to a 1:4 serial dilution of NRTIs. A minimum of 6 replicates were tested. DMSO concentrations of up to 2% had no effect on lag time or growth speed (data not shown). nd: not done.
