# Supplementary material for: Caenorhabditis elegans as a Model System for Studying Drug Induced Mitochondrial Toxicity
Source: PLoS One. 2015 May 13;10(5):e0126220. doi: 10.1371/journal.pone.0126220 (PMC4430419; doi:10.1371/journal.pone.0126220)
Supplement: S3 Table — Ct values obtained for mtDNA quantification in C. elegans exposed to AZT and fed on UV-killed E. coli. Results show no significant differences between ct values. Significance was determined using a two-sided students t-test assuming equal variance on the obtained ct values. (DOCX) [file pone.0126220.s003.docx]

**Table S3. UV inactivated *E. coli* does not alter the effect of AZT on mtDNA copy number.**

|  | Ct value | Stdev | P-value |
| --- | --- | --- | --- |
| Control | 23.67 | 0.18 | - |
| 200µM AZT | 23.04 | 0.57 | ns |
| 200µM AZT (UV treated) | 22.78 | 0.43 | ns |
